# Assessing the social validity of a brief dietary survey for Sri Lankan adults with a focus on gender: a qualitative study

**DOI:** 10.1186/s40795-021-00481-9

**Published:** 2021-11-22

**Authors:** Jessica Renzella, Santhushya Fernando, Buwaneka Kalupahana, Mike Rayner, Peter Scarborough, Nick Townsend

**Affiliations:** 1 Nuffield Department of Population Health, University of Oxford, BDI Building, Old Road Campus, Oxford, OX3 7FZ UK; 2 Sri Jayewardenepura General Hospital and Post Graduate Training Centre, Colombo, Sri Lanka; 3 Tianjin Medical University, Tianjin, China; 4 NIHR Biomedical Research Centre at Oxford and Nuffield Department of Population Health, University of Oxford, Oxford, UK; 5 Department of Health, University of Bath, Bath, UK

**Keywords:** Diet, Survey, Noncommunicable diseases, Adults, Sri Lanka, Qualitative, Social validity, User experience, Gender

## Abstract

**Background:**

As the World Health Organization urges countries to strengthen their noncommunicable disease monitoring and surveillance activities, setting-specific innovations are emerging. Diet – a key, modifiable risk factor for chronic diseases – is particularly challenging to capture reliably. By socially validating self-report dietary survey tools, we may be able to increase the accuracy and representativeness of data for improved population health outcomes. The purpose of this study was to explore the factors that impact Sri Lankan Brief Dietary Survey (a newly developed tool) and 24-h Dietary Recall participation, engagement, and social validity among Sri Lankan adults.

**Methods:**

We conducted semi-structured interviews with 93 participants (61 women and 32 men) in three Sri Lankan districts (Colombo, Kalutara, and Trincomalee). Interview data were analysed thematically and are presented as non-hierarchical thematic networks.

**Results:**

Participants identified a number of factors that influenced their survey participation and engagement. These included the time of day interviews occur, recall ease, level of commitment required, perceived survey value, emotional response to surveys, and interviewer positionality. Many of these factors were gendered, however, both female and male participants expressed a preference for engaging with socially valid research that they felt justified their personal investment in data collection. When explicitly asked to share ideas about how to improve the surveys, many participants opted not to provide suggestions as they felt they lacked the appropriate expertise.

**Conclusions:**

Our findings have implications for the accuracy and equity of dietary surveillance activities, and ultimately the appropriateness and effectiveness of programmes and policies informed by these data. Only through understanding how and why the target population engages with dietary research can we develop socially valid methods that assess and address the dietary risks of individuals and groups that are underrepresented by current conventions.

**Supplementary Information:**

The online version contains supplementary material available at 10.1186/s40795-021-00481-9.

## Background

Noncommunicable diseases (NCDs) are responsible for 73% of global deaths. Each year, 15 million people aged 30 to 70 years die from NCDs, with over 80% of this premature mortality occurring in low- and middle-income countries [1, 2]. In 2015, world leaders responded to this global health challenge by committing to reduce premature mortality from NCDs by one-third by 2030 [3]. No country is on track to achieving this goal [4]. As part of their global guidance for accelerating target achievement, the World Health Organization (WHO) has urged Member States to establish and strengthen national surveillance and monitoring systems to support setting-specific decision-making and accountability [5]. Collecting accurate and appropriate data, however, can be challenging.

Take the example of dietary data collection. Of the four behavioural risk factors heavily implicated in the development of NCDs, suboptimal diet remains the greatest contributor to NCD-related morbidity and mortality [2]. Much of this research is based on self-report measures of dietary intake, which may be subject to report and recall bias [6, 7]. Objective measures (e.g. biomarkers) can be costly, burdensome, and do not provide contextual information on what, how, and where people eat [8, 9]. While the former issues are particularly pertinent in resource constrained settings, the latter information is crucial for the development and evaluation of appropriate and effective dietary interventions in any setting. The continued reliance on self-report measures to monitor risk factors and inform policy and practice therefore requires careful consideration of a tool’s strengths and weaknesses as well as the research context and question under investigation. Not all tools are created equal, with some trading data specificity for comparability. For example, the WHO STEPwise approach to surveillance (STEPS Instrument) is a low cost and low burden surveillance method with a focus on collecting small amounts of globally comparable data [10]. This serves an important purpose but may overlook contextual detail necessary for informing national and sub-national dietary intervention development, implementation, and evaluation. Innovation is therefore required to create tools that are optimal to answer outstanding research questions in contexts with a dearth of setting-specific information.

When developing a new tool, a quantitative assessment of whether or not said tool is able to measure what it purports to measure is essential. Demonstrating quantitative validity (e.g. criterion-related), however, does not guarantee that a method is contextually-appropriate or user-friendly [11, 12]. Tool validation efforts must also consider the target population and their milieu to avoid encountering situations where groups of people and their dietary risks go unaccounted for in data collection and use [13]. Validating our work with society – by inviting participant opinions on the acceptability of and satisfaction with different research methods – is one way of illuminating where expert opinion might be at variance with user experience to the detriment of data accuracy and representativeness. In the context of intervention research, this validation process is termed ‘social validity’ [14, 15]. This present study adopts and extends the conventional definition of social validity to include research methods by inviting participant opinions on the acceptability and satisfaction with different dietary data collection methods. This extension is appropriate given that disease and risk factor monitoring and surveillance activities can and have been classed as interventions in their own right [16]. Interventions are deemed to be socially valid if their target audience believes that they appropriately address relevant issues and produce valuable outcomes [17].

Gender differences in dietary survey participation in Sri Lanka nicely illustrate the need for tool innovation and social validation. The well-documented underrepresentation of male study participation and the extrapolation of results based on female participant-dominated studies calls into question the appropriateness and effectiveness of interventions informed by these data as well as the equity implications of conventional self-report data collection methods [18–23]. Previously reported barriers to inclusive data collection – including male participant access and availability [18] – appear symptomatic of limited resources whereby female participants represent the path of least resistance to data collection. As low- and middle-income countries are encouraged to expand their national surveillance activities to capture setting-specific information, such efforts may unintentionally exacerbate gender-based inequalities.

This qualitative study is part of a wider effort to develop and validate a population-specific Sri Lankan Brief Dietary Survey (SLBDS) to assess food intake and adherence to national dietary guidelines among Sri Lankan adults [24]. The SLBDS was developed to reduce both participant and researcher burden and collect intervention-relevant data. Whilst other tools are widely (24-h Dietary Recall) and increasingly (Food Frequency Questionnaire) used in Sri Lanka, data collection and analysis burdens remain barriers to their inclusion in already cumbersome multiple NCD risk factor studies. Additionally, available evidence does not indicate that social validity or gender responsiveness considerations have informed the development or use of these surveys [7, 19, 23]. The aim of this paper is to explore the factors that impact SLBDS participation, engagement, and social validity, with a focus on gender. Participant survey improvement suggestions are also explored. We discuss the implications of these findings for future research and suggest strategies for increasing the accuracy and equity of dietary data collection in Sri Lanka.

## Methods

### Study design

Given the nature of this exploratory study that intended to understand participant views and experiences, qualitative research methods were deemed appropriate. Of the various qualitative research techniques available, semi-structured interviews were selected as the most desirable data collection method due to their ability to facilitate the exploration of underexplored topics, directed probing, and follow-up questioning [25, 26].

### Study context

Interviews took place as part of a broader validation study of a new food recall questionnaire which was powered to detect similarities and differences to an established data collection process [24]. Prior to being interviewed, each participant completed two dietary surveys, the SLBDS (a newly developed tool) and a 24-h Dietary Recall (24DR) (the reference method). The 24DR was selected as the reference method because of its wide use with the target population in national nutrition surveillance and in survey comparison and validation studies in Sri Lanka [18, 19, 23]. It takes up to an hour to administer and requires detailed knowledge to be shared and analysed. One of the aims in developing the SLBDS was to overcome the participant and researcher burden associated with administration of the 24DR.

Both the SLBDS and 24DR are structured dietary assessment tools that ask participants to recall their food and beverage consumption during the previous 24 h [24]. They differ in length, degree of survey structure, memory requirements, recall process, detail captured, and analysis burden (Supplementary file 1).

### Data collection

Between December 2018 and February 2019, we invited a purposive sample of 94 Sri Lankan adults in urban Colombo (*n* = 56), and urban and rural sectors in Kalutara (*n* = 29) and Trincomalee (*n* = 9) to participate in interviews. Having previously participated in a survey validation study that included the administration of the SLBDS and 24DR, the 94 individuals invited to participate in the study presented in this paper possessed the necessary knowledge and experience to participate in follow-up interviews. The participant recruitment method for this study was therefore the same as the quantitative validation study that preceded it [24]. This recruitment method was followed until 56, 29, and nine participants in Colombo, Kalutara, and Trincomalee were recruited. The number of participants targeted from each district was based on the population size of districts [27], as well as the amount of time that data collectors were able to spend in each location.

After participants reviewed the study information sheet and provided written informed consent for a recorded interview to take place, participant demographic data (including age, ethnicity, gender, and place of residence) were collected using open-ended questions. Self-defined characteristics are reported below. Following administration of the SLBDS and 24DR, follow-up semi-structured face-to-face interviews were conducted with each participant in their home by BK and SF (50% of the sample was allocated to each interviewer). Interviews were conducted in the participant’s preferred language (Sinhalese (*n* = 84) or English (*n* = 9)) and then transcribed verbatim into the language used by the participant. Sinhalese transcripts were then translated into English by SF. Ethics approval for this study was received from the University of Colombo and the University of Oxford. Participants were not compensated for their participation.

Participants were not required to complete both dietary surveys to be included in interviews, however, survey response rate among interviewees was 100%.

Interviewees were asked the following five questions, which were developed for this study:

1. Which survey did you prefer?
2. Why did you prefer that survey?
3. Do you have any suggestions for improving the surveys?
4. Looking at previous research, we have found that more Sri Lankan women participate in dietary surveys than men. What do you think might be the reasons for this difference?
5. Is there anything you would like to tell me that I have not asked you about?

This succinct and semi-structured question guide was developed to 1) accommodate participant and interviewer fatigue following the administration of multiple dietary surveys and 2) facilitate participant-directed discussion. Participants were not restricted by answer length or detail, and question five provided participants with an opportunity to introduce views that researchers may not have considered during interview guide development.

### Data analysis

Translated transcripts were coded by question on the basis of salient issues and themes that arose from the text. Informed by Attride-Stirling’s thematic analysis methodology, we coded for three classes of themes – basic, organising, and global – differentiated by the level of interpretation required for identification [28].

- **Basic theme**: The most self-evident theme that emerges from the data. Basic themes require the lowest order of researcher interpretation and are best understood in the context of other basic themes.
- **Organising theme:** This theme fulfils a dual function as both an aggregate of similar basic themes and a breakdown of the global theme(s). Organising themes help build a case for the super-ordinate claim.
- **Global theme**: The global theme(s) is the most abstracted from the text. It is both a summarisation and grand interpretation of lower order themes.

**Fig. 1:**
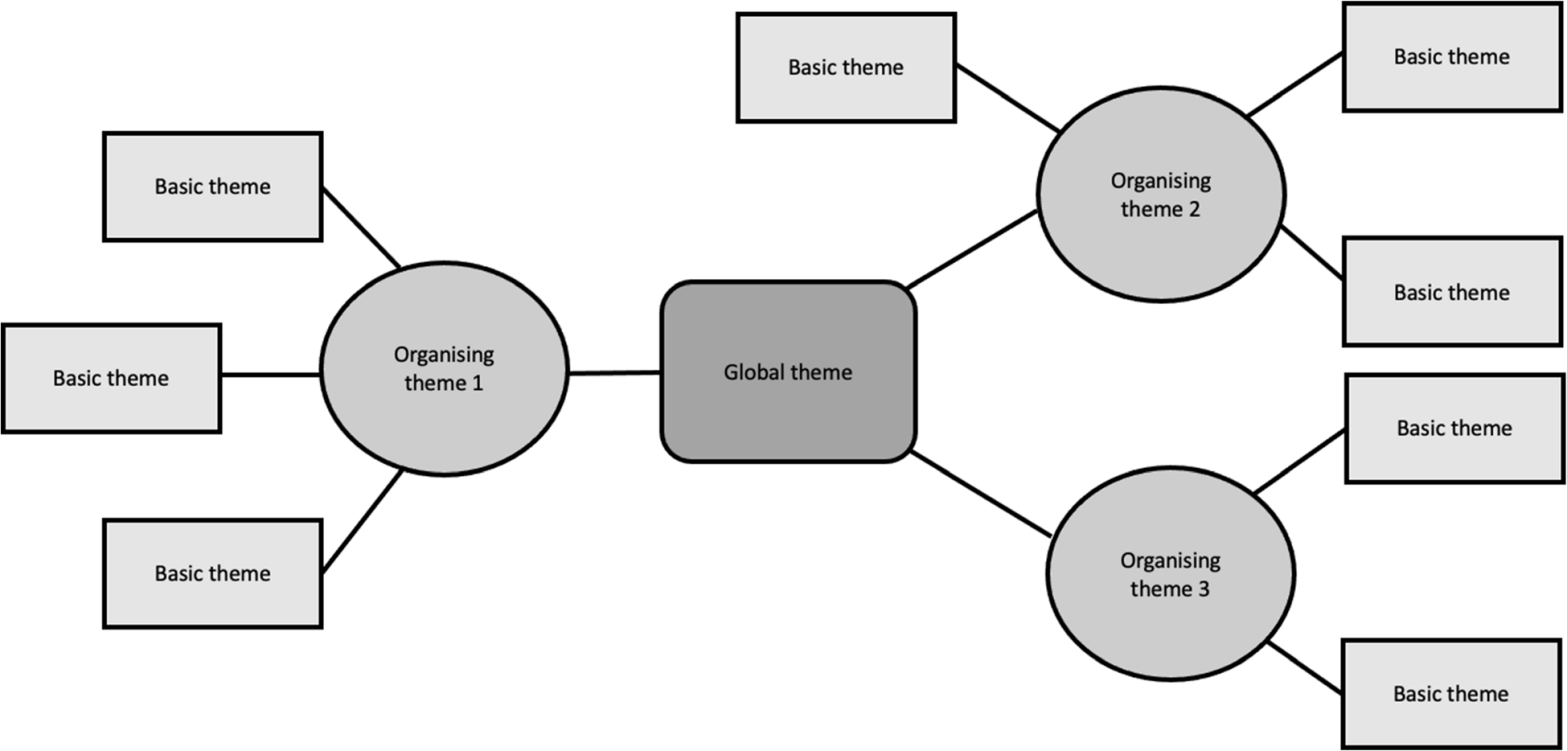
Thematic network structure from Attride-Stirling (2001)

The first author (JR), who was not present at interviews, read twice and then manually coded in Excel ten randomly selected translated transcripts covering the three districts to identify basic, organising, and global themes. The thematic groupings generated from this subset were discussed and revised with SF and NT before coding the remaining transcripts. Once all transcripts had been analysed, the wider research team discussed and revised thematic groupings. This resulted in an iterative coding and re-coding process that informed study findings and their illustration as non-hierarchical thematic network maps (Fig. 1).

## Results

### Participant profile and survey preference

Of the 94 adults invited to participate in this study, 93 provided informed consent and were included in analysis. These participants ranged in age from 18 to 65 years old *(mean = 40.7, SD = 12.6)* and resided in urban (72%) or rural (28%) sectors in Colombo, Kalutara, and Trincomalee districts. Sixty-six percent identified as female and 34% as male. Participants self-defined their ethnicity as Sinhalese (*n* = 79), Tamil (*n* = 7) or Muslim (*n* = 7) and seven individuals reported adhering to vegetarian or ‘special’ diets. A summary of participant characteristics, by gender, is presented in Table 1. When asked which of the two surveys (SLBDS or 24DR) they preferred, 37% of participants indicated a preference for the SLBDS whereas the majority (58%) preferred the 24DR. Five people *“liked both equally”* and reported no survey preference. The duration of time required to ask and respond to the five interview questions outlined above ranged from approximately 5 to 10 min.

**Table 1:** Participant characteristics (*n* = 93)

Characteristic	Female participants (*n* = 61)	Male participants (*n* = 32)
	n	
Age group (in years)		
18–29	16	6
30–44	21	10
45–59	17	13
60+	5	2
Information not provided	2	1
Ethnicity (self-defined)		
Muslim	5	2
Sinhalese	53	26
Tamil	3	4
District		
Colombo	39	17
Kalutara	19	10
Trincomalee	3	5
Sector		
Urban	45	22
Rural	16	10
Vegetarian		
Yes	1	1
No	60	31
Adheres to special diet		
Yes	2	3
No	59	29

Analysis of interview transcripts identified three thematic networks.

### Participant experience determines survey preference

**Fig. 2:**
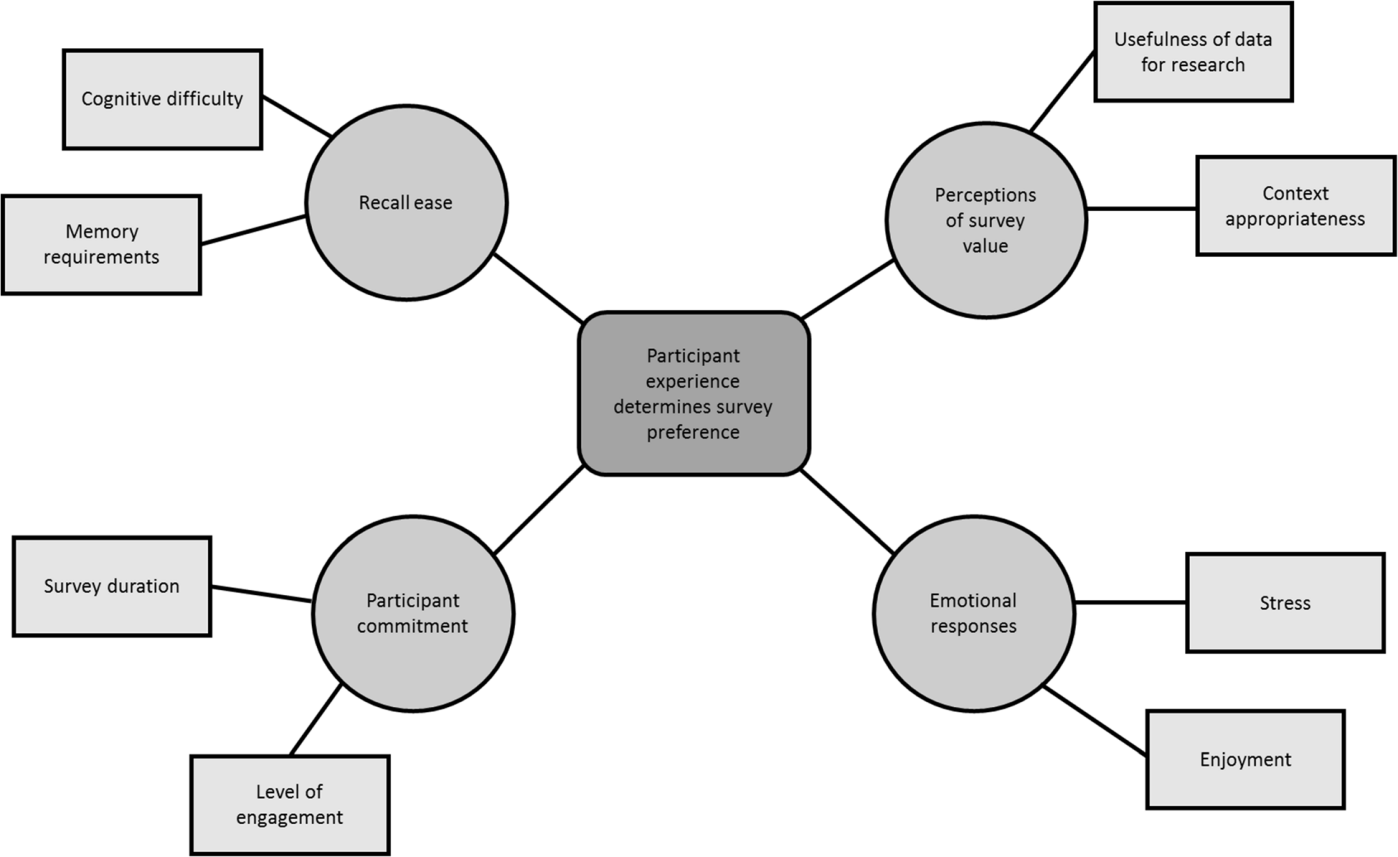
Thematic network for ‘participant experience determines survey preference’

Participant experience was the overarching determinant of survey preference (Fig. 2). The most salient themes that contributed to this finding were recall ease; participant commitment to data collection; perceptions of survey value; and emotional responses to reporting food intake.

#### Recall ease

Recall ease, a combination of the cognitive effort and memory requirements participants felt were necessary to complete the survey, was the most commonly mentioned factor that contributed to survey preference for both female and male participants. The majority of participants preferred the 24DR as they found it was easier to recall food consumed as meals: *“It was easier for me to recall meal by meal. When I answered the short questionnaire I totally forgot the herbal drinks that I consumed” (Male participant, Colombo)*; portions consumed in their own words: *“It is hard to recall in those quantities - cups, fist fulls etc.” (Female participant, Colombo)*; and 24-h intake chronologically: *“It is easier to recall the food in the order in which it was eaten throughout the day” (Female participant, Kalutara)*. A minority, however, welcomed the prescriptive reporting measurements and structured inquiry employed by the SLBDS as it prompted them to recall both what and how much they had eaten with minimal effort on their part. A few participants also felt that the prescriptive nature of the SLBDS assisted their recall of often forgotten food and beverage items (for example, condiments, snacks, and drinks): *“It prompted me to remember some of the food items I may have otherwise forgotten to recall” (Male participant, Colombo).* The level of difficulty experienced by participants in calculating, converting, and reporting their food consumption factored into survey preference decisions for both the SLBDS, *“I didn’t have to think too much” (Female participant, Colombo),* and the 24DR, *“The 24-hour Dietary Recall was much easier because it was difficult for me to convert the amounts eaten to portions in my mind” (Female participant, Kalutara)*.

#### Participant commitment

The amount of participant commitment required by each survey was a common survey preference factor for both female and male participants. Some participants preferred the *“less time consuming”* survey because they *“didn’t have to give more information than necessary” (Female participant, Colombo)* whereas others *“appreciate [d] the opportunity to give descriptive details” (Female participant, Colombo)* about their cooking methods and food intake. The SLBDS’s shorter administration duration, which *“saved”* or “*wasted less”* time, was the most commonly cited reason for preferring the SLBDS.

#### Perceptions of survey value

For some participants, the survey experience was valuable and worth their time and efforts if they perceived the data collected to be useful for diet-disease research. Seemingly contradictory statements made by the same individuals, *“I like the short survey”* and “*I think the descriptive details that can be collected [with the 24DR] will be more useful for the researchers” (Female participant, Colombo)*, illustrate nicely the survey preference thought process that resulted in them favouring the tool with ‘greater common good’.

For some participants, survey value was based on whether or not they felt that they, through their diet, were accurately represented and therefore valued in data collection: *“The short questionnaire was not representative of the way and what I eat” (Male participant, Colombo)*. Others were concerned about the appropriateness of the SLBDS in different socioeconomic contexts and how this might impact data collection and representativeness: *“The short dietary questionnaire requires you to recall the food consumption of the previous day, do the mental calculation of portions and report it. That may not be practical for less educated people” (Male participant, Trincomalee)*. The former value judgment was a feature of male participant responses whereas the latter was important to male and female participants alike. In both cases, participants preferred the more inclusive 24DR.

#### Emotional responses

Only a few female participants reflected on their preferred survey experience as being relatively *“more enjoyable”* or *“less stressful”* to the alternative. For those who associated cooking with feelings of joy or pride, the open-ended 24DR was more enjoyable as it offered an opportunity to discuss subject matter they understood and valued in detail: *“I liked that questionnaire because it asks about the dishes and what was contained in each food in detail. All this is information about the food that I myself cook. Therefore, it was more enjoyable to answer the 24-hour Dietary Recall” (Female participant, Kalutara).* The converse was true for participants who specified not being intimately involved with household cooking: *“The 24-hour Dietary Recall is asking for lengthy details that I don’t know how to answer as I don’t cook!” (Female participant, Trincomalee).* For these individuals, being asked to provide information beyond the scope of their knowledge was a more stressful experience.

### Facilitators and barriers of co-creating research methods

To involve end-users in the development of the SLBDS, participants were invited to suggest survey amendments and improvements. Some participants were forthcoming with actionable suggestions whereas many more expressed feelings of disempowerment that prevented them from sharing their ideas: *“I don’t think I have enough knowledge to suggest changes” (Female participant, Kalutara)*. Interestingly, prior demonstration of a participant’s ability to identify and articulate a need for survey improvements, either by expressing a clear survey preference (in response to interview question 1) or citing a survey’s inadequacies and less desirable features as determinants of their preference (in response to interview question 2), were not predictors of a participants’ willingness or perceived ability to suggest improvements to researchers when explicitly invited to do so.

**Fig. 3:**
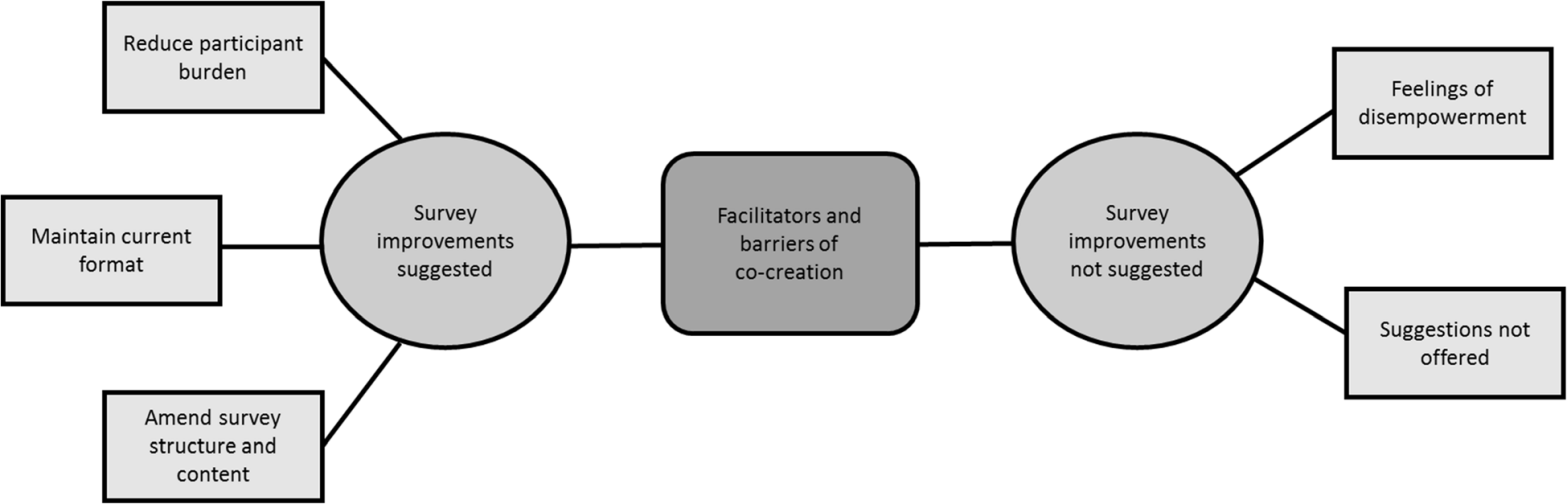
Thematic network for ‘facilitators and barriers of co-creation’

Interview question 3, “Do you have any suggestions for improving the surveys?”, had the highest non-response rate of the questions asked. In addition to participants who expressed feelings of disempowerment, half of the study sample declined the opportunity to share improvement suggestions. Figure 3 illustrates the various barriers to and facilitators of co-creating dietary survey tools with study participants.

Participants who did suggest survey improvements either expressed their satisfaction with the surveys in their current form, suggested structural and content amendments, and/or recommended strategies for reducing participation burden (Table 2). Only male participants spoke to the latter. Many of the participants who offered suggestions transformed their previously expressed 24DR and SLBDS likes and dislikes into improvement suggestions, whereas others used this explicit opportunity to engage in survey development to share insights not previously expressed in response to other interview questions.

**Table 2:** Survey improvement suggestions and representative quotations

Survey improvement suggestions	Representative quotations
Revise survey structure	*“I think dietary questionnaires should have less ‘yes’/‘no’ questions. They should be more open ended so that you can obtain richer data” (Female participant, Colombo).* *“The short questionnaire can ask the same thing meal by meal” (Female participant, Colombo).*
Expand survey content	*“There should be more detailed questions on oil consumption” (Female participant, Colombo)* *“If you asked about alcohol consumption you may be able to get a better idea of the calorie intake” (Female participant, Colombo).*
Reduce participation burden	*“[Researchers] must categorise the data into food groups after obtaining the food data as meals. When you ask a participant to recall what he ate as a food group you are trying to get him to do your job” (Male participant, Colombo).* *“Better to make these questionnaires self-administered and let the participant fill” (Male participant, Trincomalee).* *“It is very important to develop questionnaires that will not consume so much time. If not, people do not have the time to waste on things like this” (Male participant, Colombo).*
Maintain current format	*“I don’t think they need improvement. Especially the short diet questionnaire [which] adequately captures the Sri Lankan ways of eating” (Male participant, Colombo).*

### Dietary data collection is gendered

**Fig. 4:**
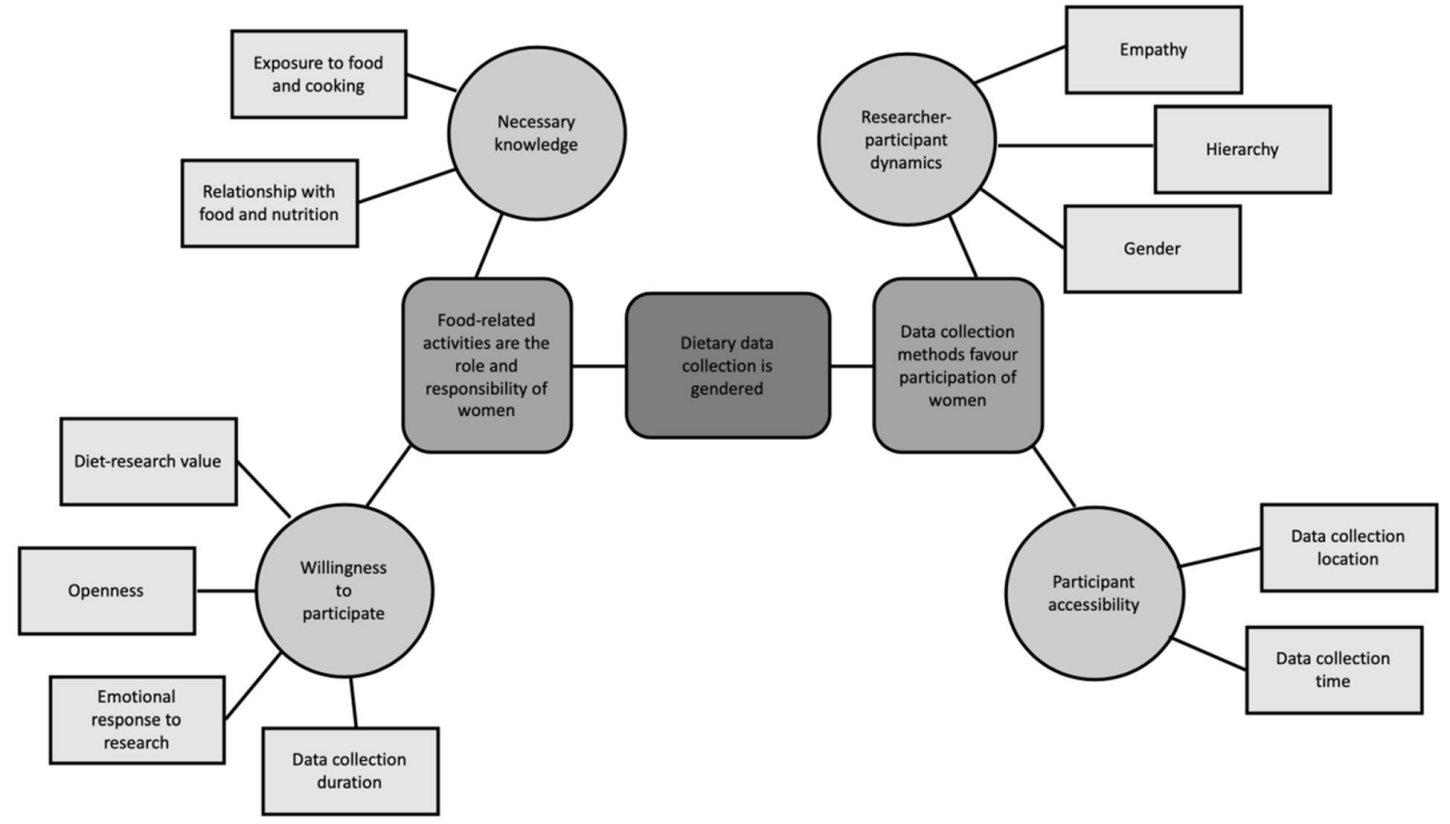
Thematic network for ‘dietary data collection is gendered’

Overwhelmingly, participants felt that both diet as a research topic and the methods used to investigate it favour the inclusion of Sri Lankan women, with implications for survey participation, level of participant engagement, and data collection burden. The thematic network for ‘dietary data collection is gendered’ illustrates the multiple themes that support and explain this claim (Fig. 4).

Female and male participants suggested that Sri Lankan women are both more willing to participate in dietary research than men and have a greater amount of necessary survey knowledge to engage. High food and cooking literacy, however, were not always spoken about as something to be revered, but rather part and parcel of being a woman in Sri Lankan society: *“There is this thing that is said here. It is not very nice. A woman’s knowledge or wisdom is the length of a kitchen spoon handle” (Female participant, Colombo)*. Some female participants spoke of possessing this type of knowledge as a burden in the context of dietary research, as it further extended the already expansive remit of women’s food-related roles and responsibilities: *“In the male dominant Sri Lankan culture everything related to food is viewed as the sole responsibility of the woman” (Female participant, Colombo).*

Despite agreeing on the existence of a clear gender-based knowledge divide, female and male participants offered varying perspectives as to how the type of food-related knowledge queried by dietary surveys is predominantly acquired by women. According to most female participants, their knowledge develops through daily exposure to “*all matters related to food, cooking, and preparation” (Female participant, Kalutara)* because *“it is the women in Sri Lanka who do most of the cooking. The men usually just eat whatever is cooked. Here, the women know the recipes [and] know how to cook so they are more knowledgeable” (Female participant, Colombo)*. Male participants who shared a view on the subject believed that a woman’s intimate relationship with food and nutrition through the life-course – *“[Women] go through pregnancy, childbirth, breastfeeding. Their nutritional requirements change in the life-cycle so they think more about food” (Male participant, Colombo)* – and relationship with body ideals and weight – *“Women are much more body conscious and think about weight and food a lot” (Male participant, Kalutara)* – were unique gendered experiences and sources of surveyed knowledge.

Female and male participants also offered differing insights as to why women might be more willing and men less so to participate in dietary research. Some female participants felt that they placed a greater value on the outcomes of diet-disease research and were therefore more willing to invest their time and effort in data collection: *“Women are more health conscious and wouldn’t mind spending time on taking part in a survey that may ultimately contribute to society” (Female participant, Colombo)*. Other female participants also described feeling valued by and enjoying research interested in a core component of their identity: *“For Sri Lankan women, food is a part of our lives (Female participant, Colombo)* so *“women like to be involved in things related to food and food research and like participating in the interviews” (Female participant, Colombo)*. Female participants generally believed that men felt quite the opposite: *“They think that answering questions about food, kitchen, etc. are beneath them” (Female participant, Colombo)* and *“also probably think that surveys such as these are not important or serious” (Female participant, Colombo)*. Some female participants suggested that men’s less open and expressive social conditioning is a primary deterrent to their participation in face-to-face and/or interview-based research that investigates personal matters, food-related or otherwise: *“I think men in our society are not very expressive” (Female participant, Colombo)* and *“They are not encouraged to engage with others about food or anything too personal about themselves. They are uncomfortable sharing things that are personal details” (Female participant, Colombo)*. According to some male participants, Sri Lankan men might be less willing to participate because the data collection process involves disclosing personal and sometimes embarrassing information about the consumption of unhealthy diets: *“Men are out and about more than women in Sri Lanka, and they may consume more junk food. Therefore, they may be reluctant to report such eating patterns” (Male participant, Colombo)*. The perceived incompatibility of long survey duration and men’s busier lifestyles was also described by some male participants as a barrier to participation: *“I think the men are generally busier than women. They don’t have the time to commit to answering long questionnaires” (Male participant, Kalutara)*.

#### Data collection methods favour the participation of women

Female and male study participants alike suggested that the administration of home-based surveys during business hours meant that women were more likely than men to be accessible to researchers: *“These surveys happen during the day. The men are out working during the day. So, women are available for answering” (Male participant, Kalutara)*. Despite consensus acknowledgement of this dual participation facilitator for women and participation barrier for men, some participants did not feel that this was a simple problem with a simple solution. One participant explained that it was in fact men’s mindset towards participation – “*men just think they have more important things to do like earning a living rather than taking part in such surveys” (Female participant, Colombo) –* that may pose the greatest barrier to their accessibility and availability, even if survey time and location were to change.

Participants also suggested that researcher-interviewee dynamics within specific information sharing dyads (for example, same or mixed gender) may foster or hinder levels of survey participation and engagement. Male participants responding to female data collectors was described as being less conducive to the participation and engagement of men whereas a data collector who is also a doctor, irrespective of their gender, might encourage male and female participation alike: **“***They don’t like to answer to females, who typically collect data” (Female participant, Colombo)* but *“if it is a medical person, anyone would be very cooperative” (Female participant, Colombo)*. Some female participants described feeling more empathetic toward female data collectors and wanting to assist them in doing a good job: *“we are more sociable and enterprising … keen to help a researcher trying to do her job. Men are less sympathetic and would not waste their time on others” (Female participant, Colombo)*. Male participants suggested that women may experience and react to researcher-researched relationship dynamics differently to men – *“I think it is easier to convince women to take part in surveys. Women are more compliant” (Male participant, Colombo)* – contributing to their expressed willingness to participate.

## Discussion

Our findings suggest that the methods most commonly used to investigate diets in the Sri Lankan context – home-based face-to-face interviews conducted during business hours – are gendered, with implications for survey participation, level of participant engagement, the social validity of methods, and the data collection burden experienced by participants. This study supports the observation that the recruitment of women represents the path of least resistance for data collection because face-to-face surveys that query diet detail simultaneously accommodate and benefit from women’s knowledge, accessibility, style of communication, and desire to ‘help out’.

By analysing participant survey preference, suggested improvements, and reflections on gender differences in participation, we gain an understanding of the barriers to and facilitators of gender-responsive survey development and the increased participation of men. Consideration of the following set of questions through a gender-sensitive lens during the survey and study development/improvement/adaptation process might therefore help to increase the representativeness of data collected (by the SLBDS and other surveys that employ similar methods), and reduce the potential negative implications of gender-based participation burden:

1. **Survey content:** Do participants have the necessary survey-relevant knowledge to participate? Are questions asked in a way that allows participants to share this knowledge?
2. **Data collection methods:** How, where, and by whom are data collected? Are these methods conducive to accurate information sharing?
3. **Social validity:** Do participants consider survey content, methods, and use to be socially valid?

### Survey content

Survey content is a combination of the information questioned and the way it is queried – both of which impact a participant’s ability to answer survey questions accurately. In this study, participants suggested that women have a greater amount of food knowledge that would favour the reporting of detailed responses. Both female and male participants found it easier to report intake as consumed throughout the day as opposed to calculating aggregate intake of specific food and beverages in prescribed quantities. Male participants more often commented on the length or time-consuming nature of the survey but both female and male participants agreed that time-efficient methods were more desirable. This conflicted with requests for the inclusion of additional questions to make surveys more representative and useful for diet-disease research. The observation that commonly used dietary surveys do not cater for diverse dietary knowledge and reporting style preferences has implications for excluding people less intimately engaged with food preparation and cooking who might therefore consume less healthy diets [29]. The SLBDS addresses participant concerns about time-efficiency but does so by collecting a focussed amount of dietary detail in a way that was not deemed socially valid by many participants. Born from a desire to reduce analysis burden, the prescribed reporting units made it more difficult for participants to share their knowledge. This aspect of the survey might need to be revisited to include multiple reporting options. The development of a comprehensive, standardised conversion guide for researchers analysing the dietary intake of Sri Lankan adults would make inclusion of this feature feasible.

### Data collection methods

#### How are data collected?

Interviews are unnatural social situations [30]. Face-to-face data collection can force a level of intimacy that study participants believed was less appealing to Sri Lankan men who may not be as comfortable sharing personal details and/or unhealthy eating habits. The increased anonymity of telephone interviews or self-administered (paper-based or online) surveys could help foster disclosure of personal or *“embarrassing”*, as one participant described unhealthy dietary habits, information. Virtual methods can also be more time efficient, produce comparable quality data to face-to-face interviews, and allow for greater data collection coverage over a wider geographical area [31]. They may, however, present a barrier to helpful and dynamic dialogue between researchers and participants (for example, tailored explanations of specific instructions) particularly useful for participants with lower diet/research literacy and experience.

#### When/where are data collected?

The time and location of data collection has implications for gendered participation. There was overwhelming agreement among participants that home-based surveys conducted during business hours favour the participation of women and reduce men’s ability to participate. Inclusive strategies to minimise the impact of these barriers might include the collection of data outside of business hours, during weekends, or in workplaces. If, however, research activities are in competition with leisure time or work commitments, they will require a high level of social validity or strategic buy-in from participants and employers. The implications for research resources to support novel data collection approaches will also need to be considered.

#### Who is collecting data?

Our study implicates diet as a research topic that is sensitive to ‘sex of interviewer effects’ [32], which in the Sri Lankan context, can be moderated by other dimensions of a researcher’s positionality. Interviews are the generation of data between two people; different dyads produce different data. Certain elements of the data collection process (for example, standardised survey tools) can be controlled, within reason, to reduce researcher influence on data production [33]. Our findings show that with a contextually-grounded, gender-sensitive understanding of the research environment, the impact of researcher-researched gender and power dynamics can also be managed to support accurate data sharing. Many researchers who match the gender of the interviewer to the gender of the interviewee (i.e. conduct ‘same-gender’ interviews) base this decision on the presupposition that rapport is more easily achieved under these conditions [34]. The reality, however, is not that simple. Participants in this study identified women interviewing women and doctors (irrespective of gender) interviewing both women and men as conducive for sharing reliable data. This study did not, however, investigate the vulnerability of different participants to various power imbalances and the associated ethical implications of this. Comments offered by few male participants that *“women are more compliant”* and *“easier to convince”* indicate that further research is needed to understand whether certain participants might feel pressured to participate in dietary research activities in the Sri Lankan context.

### Social validity

Both female and male participants expressed a preference for engaging with socially valid research that they felt justified their personal investment in data collection. Study participants tended to appraise social validity by asking ‘*Am I valued in this research?’* or ‘*Is this research valuable for society?’*. If the answer was *‘yes’* to either, then the participant associated the survey with a useful expenditure of their time and effort. The prescriptive SLBDS was deemed more exclusive with respect to what it included and therefore who it included. Survey content amendments and better communication with participants about the research purpose and participant value may ameliorate some of these exclusive/exclusion concerns. Ideally, more effective communication and tailored study information would be informed by a more detailed understanding of the different value-based motivations for participation and engagement. Future research could further support this endeavour by exploring how the intersectional relationships between participant age, district of residence, ethnicity, and gender might impact survey social validity.

#### Co-creation

Under the presupposition that co-creating research methods with end-users improves method acceptability and satisfaction [15, 35], we invited and analysed participant feedback and survey improvement suggestions. From the responses and reactions of participants, many of whom expressed feelings of disempowerment that precluded them from sharing their ideas with an ‘expert’, we learnt that a greater level of context-appropriate attention and planning needs to be invested in creating conducive conditions for participant engagement in the co-creation process. Further research is required to understand what successful co-creation with research participants in Sri Lanka might look like.

### Comparison with other literature

Gendered physical survey participation barriers (for example, access and availability during work hours) are well-documented in the Sri Lankan dietary assessment literature [18, 20]. Other explanatory factors have not been explored. Our findings support the known barriers and illuminate additional features of conventional data collection methods that favour the participation of women. The finding that people, irrespective of gender, prefer shorter and easier surveys is not a revelation, and reflects the trend of converting dietary assessment tools into brief versions [7]. This does however conflict with participant desires for more expansive surveys that represent diverse populations and diets. Neither the Sri Lankan literature nor this study explore conflict resolution strategies that weigh different aspects of participant preference that may impact survey social validity. Previous studies have suggested that different research topics and contexts favour different combinations of same- and cross-gender interview pairings [32]. For example, in contexts where men more often discuss intimate topics with women, cross-gender dyads have been preferred [34, 36]. In the Sri Lankan context where men are described as generally *“less expressive”,* it is difficult to know which pairing(s) would generate greater rapport. Our study does not provide detailed insight on this topic; participants did not discuss whether men would be more comfortable being interviewed by men and we have no comparison data (female researchers conducted all interviews). To the authors’ knowledge, this is the first study to explore the social validity of dietary data collection tools for Sri Lankan adults. The global dietary assessment literature is also thin when it comes to discussing social validation, although various ‘acceptability’ assessments (both quantitative [37] and qualitative [38]) do form part of some survey development and validation studies. Comparatively, the social acceptability and importance of health interventions (goals, procedures, and outcomes) are an established and expanding area of research [15]. Increasingly, NCD researchers are being encouraged to assess the social validity of health interventions, programmes, and policies – as a moderator of effectiveness – but are not extending a similar level of inquiry to the data collection tools used to inform such activities [17]. This highlights an important inconsistency and begs the question: can a programme, policy or intervention achieve social validity if it is not informed by tools subject to the same assessment criteria?

### Strengths and limitations of this study

The main strengths of this study are the large sample size and the use of semi-structured qualitative research methods that allowed participants to direct discussions and share emic perspectives. A key limitation of this study is that it only involved three districts in Sri Lanka, which limits the generalisability of findings. Our research also suffers from the very phenomenon it is trying to explore – low participation of men in dietary research. This implicates our findings in a cycle whereby the experiences and preferences of women shape the data collection process and potential propagation of its gendered nature and burden. The self-selecting participants who participated in our study may not be representative of the wider population, necessitating further research with those who would ordinarily not engage with dietary data collection. As indicated by participant experiences and the interviewer effects literature [32], the gender of the data collectors in this study may have impacted how questions were asked and answered. Without a comparison, we were unable to assess these impacts. Another potential limitation of this study is that the primary coding was conducted by one researcher. Although all codes and themes were discussed at length throughout the analysis process with the wider research team, intercoder reliability could not be evaluated [39]. Many of the interviews had also been translated from Sinhala into English, a process that *“involves converting ideas expressed in one language for one social group to another language for another social group, which entails a process of cultural decoding”* [40]. To ensure that any ‘lost in translation’ incidents were minimised, data extracts as well as codes and themes were discussed at length with members of the research team involved in data collection and transcript translation. It is also worth noting that participants in this study self-defined as either female or male but used the terms females/women and males/men interchangeably when referring to gender in their responses to interview questions. We have stayed true to participant wording/translations in this paper and the wording used by authors when referencing the Sri Lankan literature, however, the use of these terms and their meaning within the Sri Lankan context requires further investigation. It is also important to acknowledge that gender is not a binary concept, and that future studies should explore further the gendered issues reported on in this study. Finally, systematic member checking was not undertaken with participants, which would have strengthened further the credibility of results.

## Conclusion

Dietary research is vulnerable to trading on gender stereotypes. This has implications for surveillance activities, and ultimately the appropriateness and effectiveness of programmes and policies informed by these data. Many of the barriers to general and gender-specific participation and engagement uncovered in this study are not insurmountable. Overcoming them, however, requires contextually-grounded, gender-sensitive investment in data collection focussed on currently underrepresented individuals and groups. In the context of expanding dietary surveillance in Sri Lanka and low- and middle- income countries more generally, the relationship between data quantity, quality, and equity requires further setting-specific consideration. Exploring the social validity of new and existing measurement methods will assist this effort, which should be supported by the development of context-appropriate measures of social validity.

## Supplementary Information

**Additional file 1: Supplementary file 1.** Comparison of Sri Lankan Brief Dietary Survey and 24-h Dietary Recall characteristics

## Data Availability

The data generated and analysed during the current study are not publicly available as ethical approval does not extend to the sharing of participant data or interview transcripts beyond study authors.

## Abbreviations

FFQ: Food Frequency Questionnaire
NCD: Noncommunicable Disease
SLBDS: Sri Lankan Brief Dietary Survey
SLFBDGs: Food Based Dietary Guidelines for Sri Lankans
WHO: World Health Organization
24DR: 24-Hour Dietary Recall
7DWR: 7-Day Weighed-Intake Dietary Record
