# Supplementary material for: Assessing the social validity of a brief dietary survey for Sri Lankan adults with a focus on gender: a qualitative study
Source: BMC Nutr. 2021 Nov 22;7:79. doi: 10.1186/s40795-021-00481-9 (PMC8607623; doi:10.1186/s40795-021-00481-9)
Supplement: Supplementary file 1 — Additional file 1: Supplementary file 1. Comparison of Sri Lankan Brief Dietary Survey and 24-h Dietary Recall characteristics [file 40795_2021_481_MOESM1_ESM.docx]

**Table S1.** Comparison of Sri Lankan Brief Dietary Survey and 24-h Dietary Recall characteristics

| Dietary assessment characteristics | Sri Lankan Brief Dietary Survey (SLBDS) | 24-hour Dietary Recall (24DR) |
| --- | --- | --- |
| Study design | Cross-sectional | Cross-sectional |
| Time period of interest | Previous 24 hours | Previous 24 hours |
| Information queried | Specific components of diet (excluding contextual details) | Total diet  (including contextual details) |
| Survey structure and length | Structured  1 A4 page  Reporting units prescribed | Structured  Variable length  Reporting units not prescribed |
| Time required to complete | 5 – 15 minutes | 30 minutes – >1 hour |
| Recall requirements | Total consumption by food group in prescribed units | Consumption as recalled |
| Data coding and analysis | Data coding and unit conversion not required | Data coding and unit conversion required |
